# The Neurobiological Correlates of Gaze Perception in Healthy Individuals and Neurologic Patients

**DOI:** 10.3390/biomedicines10030627

**Published:** 2022-03-09

**Authors:** Simone Battaglia, Jasper H. Fabius, Katarina Moravkova, Alessio Fracasso, Sara Borgomaneri

**Affiliations:** 1 Centro Studi e Ricerche in Neuroscienze Cognitive, Dipartimento di Psicologia, Alma Mater Studiorum-Università di Bologna, 47521 Cesena, Italy; 2 Institute of Neuroscience and Psychology, University of Glasgow, Glasgow G128QB, UK; j.h.fabius@uu.nl (J.H.F.); katarina.moravkova@glasgow.ac.uk (K.M.); alessio.fracasso@glasgow.ac.uk (A.F.); 3 IRCCS Fondazione Santa Lucia, 00179 Rome, Italy

**Keywords:** gaze perception, gaze-cueing, neuroimaging, superior temporal sulcus, amygdala, neurologic patients, neurocognitive mechanisms, non-invasive brain stimulation

## Abstract

The ability to adaptively follow conspecific eye movements is crucial for establishing shared attention and survival. Indeed, in humans, interacting with the gaze direction of others causes the reflexive orienting of attention and the faster object detection of the signaled spatial location. The behavioral evidence of this phenomenon is called gaze-cueing. Although this effect can be conceived as automatic and reflexive, gaze-cueing is often susceptible to context. In fact, gaze-cueing was shown to interact with other factors that characterize facial stimulus, such as the kind of cue that induces attention orienting (i.e., gaze or non-symbolic cues) or the emotional expression conveyed by the gaze cues. Here, we address neuroimaging evidence, investigating the neural bases of gaze-cueing and the perception of gaze direction and how contextual factors interact with the gaze shift of attention. Evidence from neuroimaging, as well as the fields of non-invasive brain stimulation and neurologic patients, highlights the involvement of the amygdala and the superior temporal lobe (especially the superior temporal sulcus (STS)) in gaze perception. However, in this review, we also emphasized the discrepancies of the attempts to characterize the distinct functional roles of the regions in the processing of gaze. Finally, we conclude by presenting the notion of invariant representation and underline its value as a conceptual framework for the future characterization of the perceptual processing of gaze within the STS.

## 1. Introduction

In any social situation, the direction of one’s gaze serves as a fundamental method of communication through which individuals exchange information. Gaze can express relevant information about the mental states of others, support social control, regulate turn-taking, guide attention and communicate intimacy [1,2,3,4]. Shifting the attention to the direction in which another person’s attention is oriented is associated with the gaze-cueing of attention [5,6,7]. The direct gaze of others means that attention is directed to the observer, while averted gaze implies that the attention of the other is directed to the environment; consequently, averted gaze may also cause the observer to make reflexive shifts of attention toward the environment [8]. The behavioral index of such a joint shift of attention is called the gaze-cueing effect [9] in which human observers have faster saccadic or manual reaction times (RT) to objects appearing at the gaze-congruent locations compared with objects presented in gaze-incongruent locations [9,10,11].

Gaze-cueing is considered an involuntary and reflexive effect (see [12,13] for a review), and it is assumed to occur rapidly due to the pivotal role of gaze as a social and biological trigger [14]. However, gaze-cueing is not an encapsulated effect, as it seems to be permeable to contextual influences carried by the face [15], such as facial expression [16,17,18,19] and social (e.g., familiarity) or perceptual (e.g., dominance) characteristics [10,20,21]. It is crucial to mention that within a neuroimaging context, it is possible to investigate the neural bases of gaze-cueing (i.e., the fMRI contrast involving averted and direct gaze observation, in the presence of a concomitant behavioral task to measure the gaze-cueing effect) and the neural correlates of gaze direction (i.e., the fMRI contrast between averted and direct gaze observation in the absence of a concomitant behavioral task to measure the gaze-cueing effect). Similarly to *gaze-cueing* in behavioral studies, the perception of *gaze direction* and contextual information can interact [22,23,24]: when emotion and gaze direction are congruent (e.g., an angry expression with a direct gaze), the perception of that emotion is facilitated, but when emotional expression and gaze direction are incongruent (e.g., an angry expression with an averted gaze), emotion perception is impaired.

From a neuroimaging perspective, there are still unanswered questions about gaze-cueing and gaze direction: (i) What are the neural correlates that mediate gaze-cueing and the perception of gaze direction? (ii) Are the neural bases of gaze-cueing and the perception of gaze direction affected by contextual factors, such as emotional expressions?

**Figure 1:**
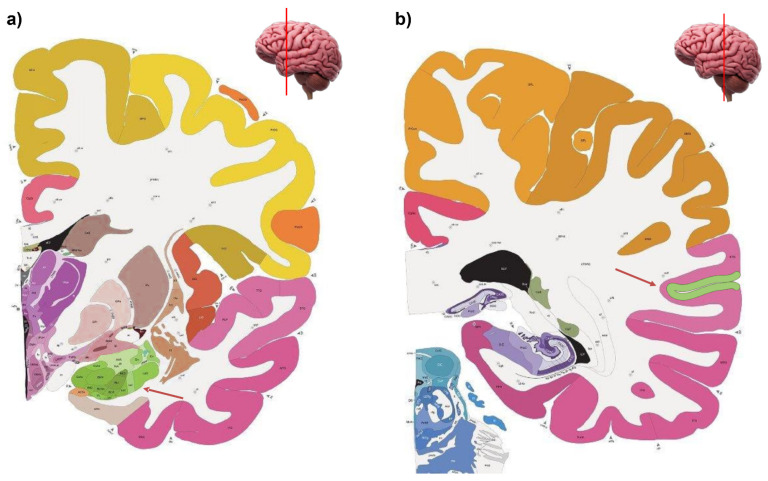
Coronal section of the brain in which the amygdala (panel **a**) and the supe-rior temporal sulcus (panel **b**) are colored in green. The red arrows are oriented towards the neuroanatomical structure of the amygdala and the superior temporal sulcus respectively. The representation of the coronal section of the brain in the present figure was generated free of charge using the Allen Brain Atlas (https://atlas.brain-map.org/, accessed on 24 February 2022).

Potential neural candidates for the visual processing of others’ gaze that could allow for interactions with social factors (such as emotional expressions) are the superior temporal sulcus (STS) and the amygdala (see Figure 1).

The STS is part of a distributed neural system for facial information processing, together with the inferior occipital gyri and fusiform gyri [25]. The amygdala is responsive to emotional facial expressions [26,27,28]; thus, it is a likely candidate for the contextual modulation of face processing. It was reported that the STS is required for the encoding of gaze information [8,29,30]. Notably, the role of the amygdala in gaze processing was reported by several neuroimaging studies [8,31]. This may indicate that the amygdala has a broader role in attentional processes besides its role in emotion processing. In particular, it was argued that the amygdala is activated when the gaze signals a possible threat, as expressed by angry and fearful faces [32,33,34,35]. However, despite their mutual involvement in gaze processing, the amygdala and STS respond differently to emotion-related faces and to the concomitant presence of gaze and emotional expression. It is worthwhile to understand the neural bases of gaze direction and gaze-cueing and which contextual factors, such as facial emotional expressions, can modulate the subsequent brain activations. In turn, this helps with understanding the underlying processes causing impairments in social cognition and social functioning that are associated with various psychiatric, neurological and neurodegenerative illnesses [36,37,38,39,40].

Here, we investigated the close interaction between gaze perception and other changeable features of the face (e.g., emotional expressions), along with the neural systems underlying gaze-cueing and the processing of gaze direction. Although other areas were found to be implicated in gaze processing (e.g., fusiform gyrus and even frontal areas), we focused our review on the role of the STS and the amygdala since our main goal was to shed light on their roles in integrating gaze perception and emotional expressions. We concluded our review by showing data of brain-damaged patients and non-invasive brain stimulation evidence, highlighting the crucial roles of the STS and amygdala in gaze perception.

## 2. Neural Correlates of Gaze Direction: The Pivotal Role of the STS

The close interaction between eye movement, gaze perception and other changeable features of the face, such as emotional expressions, was demonstrated using neuroimaging and brain stimulation techniques, as well as in studies of brain-damaged patients. In this section, we discuss recent neuroimaging evidence investigating the visual processing of gaze-cueing and gaze direction. As mentioned in the introduction, likely neural candidates for visual processing of others’ gaze direction that could allow for interactions with social factors are the superior temporal sulcus (STS) and the amygdala.

Neuroimaging studies showed that amygdala activation is not receptive to gaze direction per se [41], but instead is implicated in the emotional processing of gaze information [42,43]. This interpretation can explain why the amygdala shows differential activations when comparing the observation of direct versus averted sight [44]: some studies using neutral facial expressions reported that the amygdala was more active for direct than averted gaze [45,46], where this can be explained by the higher saliency of the direct gaze, which can be considered as a frequent trigger for social interaction, while others, using a social task (i.e., attribute hostile or friendly intentions to actors with a direct or averted gaze), found the opposite [47,48], indicating that the averted gaze may be more informative in an emotional context. Finally, other researchers reported no evidence of amygdala activity in response to gaze direction [49].

In terms of a functional difference between the STS and amygdala, it was suggested that the attentional shifting toward a joint object is a critical function of the STS, while the amygdala may appear to be required for combining emotional expression and gaze direction [32,50,51] rather than in the perception of gaze direction per se [41]. Supporting the role of STS in gaze perception, a seminal fMRI study [52] employed a one-back task in which participants attended selectively to the gaze direction or the identity of each face (match-to-sample task). Results revealed that the perception of gaze direction was mediated by regions in the STS, and it was distinct from the inferior occipital and fusiform gyri, which are dedicated to the perception of face identity. These results are compatible with the concept proposed by Haxby and colleagues [52] (also see [53]), indicating the posterior superior temporal sulcus (pSTS) as one of the three occipitotemporal regions attributed to visual face analysis (specifically, gaze perception), together with the inferior occipital gyrus (IOG) [54] and the lateral fusiform gyrus (lFG) [55]. The STS was also highlighted using a paradigm where participants were adjusted to gaze direction [56]. These authors measured blood oxygen level dependence (BOLD) by means of fMRI adaptation to gaze redirected to the left or right relative to the test faces with straight gazes. The authors found BOLD response suppression in the anterior STS and inferior parietal lobule (IPL), especially for faces with gaze averted to the adaptor’s side. In 2003, Pelphrey and co-workers [57] observed shorter hemodynamic responses when participants viewed faces gazing toward objects (in this case, a checkerboard in the visual field) compared with faces gazing toward empty space. This pattern of responses was seen in the right STS, intraparietal sulcus and fusiform gyrus, supporting the assumption that the context in which an eye movement occurs affects brain activity associated with gaze shift perception [58,59].

An interesting study by Carlin and colleagues [60] helped to further clarify the role of the STS in response to faces with different gaze directions. These authors showed that the (bilateral) anterior part of the STS is implicated in the processing of gaze direction. Crucially, they demonstrated that gaze direction sensitivity in the anterior STS was independent of head orientation. These findings indicate the involvement of this area in the view-invariant representation of another’s social gaze direction, suggesting a hierarchical processing stream for gaze perception with increasing invariance to gaze-independent features (head orientation) from the posterior to anterior STS [61].

Together, the studies mentioned here revealed that the STS has a pivotal role in processing the direction of gaze compared to the amygdala, at least when neutral facial expressions are presented.

## 3. Difference between Gaze Cues and Other Symbolic Cues

Multiple neuroimaging studies compared the neuroanatomical correlates of joint attention conveyed by gaze shifts to the correlates of attentional shifts induced by other symbolic cues without using face stimuli. These studies addressed the important question of the specificity of the STS to facial stimuli in the context of gaze perception. Previous studies investigated two alternatives: (i) Is the STS specialized for processing social attention (such as gaze cues)? (ii) Is the STS more broadly processing any symbolic cue (social or not)? In the following section, we summarize papers that investigated the specificity (or lack thereof) of the STS in the social orienting network. We identified recent studies comparing gaze and symbolic cues in fMRI data [42,62,63,64,65,66,67,68,69,70,71,72]. We first summarize their findings and then synthesize them. In agreement with the idea that the STS may be sensitive to intentions expressed by eye gaze [57,73], Hooker and colleagues [42] showed a specificity of the STS in response to gaze cues. The authors showed that gaze and arrow cues recruit distinct brain regions, with gaze cues preferentially activating occipito-temporal regions and arrow cues enhancing the activation of occipito-parietal regions. Importantly, relative to arrow cues, gaze cues showed increased activation in the human FFG and the STS. Furthermore, Lockhofen and colleagues [62] investigated the differences in the activation patterns associated with incongruent gaze and arrow cues in a gaze-cueing task, with a primary focus on brain regions that are involved in the processing of social information (i.e., the STS and fusiform gyrus). Their behavioral data showed that congruent stimuli produce a faster reaction time and that this improvement is more significant with non-social cues, while their imaging data were in line with other reports showing that the STS is more sensitive to gaze shifts than to directional arrow cues [42,63]. This study additionally showed that, unlike arrow cues, the STS did not reveal increased connectivity with the amygdala for gaze, but the connectivity was enhanced between the fusiform gyrus and the amygdala. Similar results were observed in 2008 [64] in which the authors, focusing on specific ROIs in the STS and IPS, indicated the role of the STS in processing eye gaze in a relevant social context. The authors found that gaze cues were associated with bilateral activations in the pSTS region and the cuneus (also see [74]). Therefore, higher STS activation may indicate gaze cues’ social relevance over non-social cues. 

However, in line with the idea of a lack of differences between symbolic and gaze cues in the STS, Sato and co-workers [75] used fMRI to examine participants’ brain activity while they watched directional and non-directional stimuli (including eyes, hands and arrows), which may act as cues for a subsequent target object that could appear in a congruent or incongruent location. Behaviorally, no gaze cue was detected (i.e., the number of accurate responses or RTs did not significantly differ between the stimulus type or direction), and only eyes and arrow cues triggered activity in the right STS, while the ‘hands’ contrast was only significant using a more liberal statistical threshold. Modest amygdala activation was found specifically in response to eye direction. These results show that the amygdala might be involved in the processing of eye direction, while the STS region is considered to be active for both gaze-related functions and joint attention processes, regardless of the kind of cue that signals the shift of attention.

In addition, in 2014, Callejas and colleagues [66] tackled the question of STS specificity in gaze-cueing, probing the existence of a distinctive mechanism of attention for social cues by comparing the brain activation for social (i.e., computer-generated realistic photos of male/female faces with visible neck and shoulders) and symbolic cues (i.e., arrows), separately evaluating orienting attention in space and reorienting attention for object detection. Results showed no interaction effect between cue validity and cue type, and no evidence was discovered linking face-selective regions (i.e., bilateral fusiform face area (FFA), bilateral occipital face area (OFA) and right posterior superior temporal sulcus (pSTS)) to a hypothetical, specialized mechanism for orienting attention in response to gaze cues reflected by BOLD amplitudes. Interestingly, functional connectivity analysis revealed increased connectivity between face-selective regions and the right intra-parietal sulcus (IPS), pSTS, the amygdala and the inferior frontal gyrus (IFG) during gaze-cueing. The enhanced connectivity suggests that the attentional networks obtain gaze information from face-selective regions, which acquire them from gaze signals. In line with Callejas’ findings, Greene and colleagues [67] showed the specific recruitment of extra-striate regions in gaze-cueing (not including the STS or the amygdala). There are also reports showing that symbolic cues selectively activate the STS rather than gaze cues, hence going against the idea of the specificity of the human STS in the social orienting network. Additionally, Engell and colleagues [68] further investigated whether attentional orienting due to the gaze-direction perception and symbolic directional cues (e.g., arrows) are processed by the same brain areas. Behaviorally, both arrow and gaze cues led to faster reaction times during valid trials than during invalid trials, similar to previous findings [76,77]. Importantly, invalid gaze/arrow cues produced a significantly stronger response than valid cues in the right lateral temporal cortex, including the pSTS/temporoparietal junction (TPJ) and the right inferior parietal lobe. In fact, the arrow cues were the main factor that determined this effect. Moreover, despite similar behavioral effects and neural activations, gaze and arrow stimuli triggered the attentional systems differently: invalid arrow cues increased the hemodynamic response in the ventral frontoparietal attention network (specifically, the right TPJ and IFG) compared to valid arrow cues. Gaze cues did not display this difference. Similarly, Hietanen and co-workers [69] reported a difference in the activation of the middle temporal gyrus (anatomically close to the STS) for non-symbolic and gaze cues, with stronger activations for non-symbolic cues, although a gaze generates a significantly larger cueing effect than arrow signals. In a similar study to that conducted by Engell and colleagues [68], Joseph and colleagues [70] found similar behavioral data; however, in contrast to Engell and colleagues’ findings, they found that the contrast between invalid and valid gaze cues engages the ventral attention system, specifically, the TPJ and inferior parietal cortical nodes extending to the STS, when participants redirected their attention following the presentation of invalid gaze cues, but not after invalid arrow cues. From these data, the authors concluded that gaze direction engages the attention networks more robustly, suggesting that attentional differences between social and non-social stimuli might be quantitative rather than qualitative, contrary to existing theories, which suggest that the improved processing of social stimuli requires a specific network of brain areas [78]. Moreover, Uono and colleagues [71] performed an MEG source reconstruction analysis to examine the temporal patterns of the neural activation during attentional shifts induced by gazing and arrows. The results revealed that after 200 ms, the STS and the inferior frontal gyrus were activated only when directional cues were presented, suggesting that the brain mechanisms behind attentional shifts elicited by gazing and arrows have similar spatial and temporal distributions. The 350–400 ms time window showed different neural activations in response to gaze and arrow stimuli; however, such activation did not include the STS. Since the difference in attentional orienting between gaze and arrow cues might be distinguished only when the cues are part of a rich setting, Zhao and co-workers [72] compared gaze and arrow cues pairing social gazing and social voice or arrow and tone (congruent condition) or combining social gazing and tone or arrow and social voice (incongruent condition). The results showed that invalid conditions modulated the activity of the ventral frontoparietal network (i.e., left TPJ and IFG), while no activation differences in the cortical areas associated with attentional orienting (i.e., the STS and TPJ) were found in the contextual elaboration between gaze and arrow cues.

To conclude, while the STS is sensitive to gaze perception [25], its role in gaze-cueing is not clear. While several authors reported a specific role of the STS in processing gaze cues compared to non-symbolic cues [42,62,63,70], there is enough non-converging evidence regarding the specificity of the STS to be cautious about deriving definitive conclusions [68,69,72,75]. Indeed, it is important to mention that, even behaviorally, most of the aforementioned studies failed to show a higher cueing effect (i.e., faster reaction time for congruent relative to incongruent cues) for gaze cues compared to non-symbolic cues [62,66,68,75]. Thus, it is possible to speculate that this behavioral lack of advantage for a gaze versus non-symbolic cue is also reflected at the neural level. From the existing literature, it is not possible to disambiguate whether the human STS has a specific role in attention orienting to social stimuli or whether it encodes the visual input and passes information to a supra-modal attentional system.

## 4. Conjoint Processing of Emotional Expressions and Gaze Direction in the Amygdala and STS?

During social communication, emotional expression is useful for communicating the inner state of others and predicting their potential actions. When changes in emotional expression are associated with gaze shifts, the social cues of the other reveal information that guides individuals’ behavior toward or away from other stimuli in the environment. Gaze cues play a significant role when conveying fear, where the meaning of the emotion is ambiguous until the source of the emotional change is determined (i.e., to identify the location of the threat source). The interaction between gaze perception and emotional expression is associated with amygdala and STS activation, which is crucial regarding both facial expression and gaze perception [33,65,79]. However, the neural basis of gaze and emotional cue integration during the attentional orienting process is still unknown. In the following section, we review recent studies investigating the conjoint processing of emotional expression and gaze direction, as well as aspects of the visual presentation of stimuli that can affect the processing of facial emotions (see Table 1) [32,33,42,50,65,79,80,81,82,83,84,85,86].

Although it was not the authors’ primary focus of investigation, Hooker and colleagues [42] employed angry and happy faces in a gaze-cueing paradigm. They found that angry faces generated greater activation in the STS region than happy facial expressions. The activation was observed in an area extending from the dorsal STS to the IPS. The fact that STS activity is modulated by facial expressions strengthens the view that this area is receptive to gaze cues related to social interaction [87]. When the task was to detect an infrequent gaze direction change, increased selective amygdala activity was found. To investigate whether the amygdala is involved in the conjoint process of gaze direction encoding and facial expression processing, Adams and colleagues [32] combined the presentation of fearful/angry faces with direct or averted gaze. The authors found that the left amygdala responded strongly to angry averted and fearful directed gaze conditions, whose threat to the subject was difficult to determine. This evidence highlights the role of this neural structure in recognizing not only the presence of a facially conveyed threat but also the ambiguity associated with it. Interestingly, amygdala responses were significantly greater when anger was expressed with averted gaze, where it was only shown by cultural in-group members [80]. In 2004, Sato and colleagues [50] performed a similar experiment to Adams and co-workers. They recorded the BOLD signal while participants were presented with angry/neutral expressions facing or looking away from them. The authors focused their analysis on the amygdala and found that the amygdala was more active for angry expressions looking toward the subjects than those looking away from them (in contrast with Adam’s results). These results jointly corroborate the concept that the amygdala shows high sensitivity to the emotional expression of a facial stimulus, whereas its response to gaze direction is less clear (however, see Section 4.2 on ‘Transient or sustained presentation of visual stimuli’ for a potential account of amygdala response to gaze direction).

The conjoint processing expressions and gaze direction were explicitly studied also in the STS. An experiment was carried out by Engell and Haxby [81] that aimed at comparing the activation evoked within the STS by different emotional expressions and gaze directions. Regarding this aim, the authors showed participants with neutral faces with a direct or averted gaze, or emotionally expressive faces (e.g., anger, disgust, fear and surprise) with a direct gaze. The results showed a greater STS response when viewing emotional facial expressions as compared to neutral faces and that perceiving faces with an averted as compared to a direct gaze led to greater right STS activation. Unfortunately, the authors did not investigate possible differences across emotions in the activation of the STS. However, a further inspection of the responses within the right STS showed how expression and averted gaze activated distinct, though overlapping, cortical areas. These data are in line with clinical evidence that prosopagnosia patients can show an impaired perception of facial expressions without necessarily suffering from gaze perception deficits [88]. In 2008, Hadjikhani and co-workers [33] performed an fMRI study aiming at investigating whether fearful facial expressions with gaze averted toward a potential threat in the environment elicited more activation in brain areas involved in adaptive action planning. Fearful faces with an averted gaze altered the activity in the amygdala and STS more than fearful faces with a direct gaze. These data suggest that the gaze direction triggers the combined process of facial expression with the information given by the gaze direction. These results are in contrast with the study of Adams and colleagues [32] in which fearful faces with direct gaze were found to activate the amygdala. To further investigate the interaction between emotion and gaze as a way to shed light on the existing contrasting results [32,33,50], N’Diaye and co-workers [79] employed a controlled set of virtual animated faces, which could express emotions dynamically and with different intensities and gaze shifts directed toward or away from the participant. Their results showed that gaze direction modulated neural responses to parametrically manipulated fearful and angry expressions (i.e., by manipulating the intensity of these expressions using a morphing procedure) only when they were ambiguous (in the middle of the fearful–neutral continuum). In line with previous imaging studies, these results showed greater amygdala activation for fearful faces when their gaze was oriented away from rather than toward the observer [33,89], as well as stronger amygdala activation for angry faces looking toward rather than away from the observer [50,89]. Interestingly, the present data seem to suggest that a possible explanation for the contradictory results may be the effect of the emotion intensity on gaze-related effects. Critically, the STS was more active when faces were looking away from the observer relative to direct gaze trials (as in [81]). However, no evidence for the role of the STS in the combined perception of emotion and gaze was reported. 

Furthermore, to investigate whether individual differences in anxiety levels may explain the abovementioned inconsistent findings, Ewbank and colleagues [82] examined the effect of gaze direction on amygdala activation for the facial expressions of anger, fear and neutral expressions, controlling for differences in individual anxiety levels. They found that angry faces evoked greater responses in highly anxious participants compared to angry faces with an averted gaze, while the response to fearful faces increased with anxiety for both direct and averted gaze conditions. Recent studies untangled the pathophysiology underlying anxiety disorders: experimental and preclinical evidence revealed that anxiety disorders are determined by the abnormal neural processing of threat-related stimuli, which is mediated by the cyclic AMP (cAMP)–protein kinase A (PKA) pathway [90,91,92]. Additionally, the downregulation of the regulatory subunit of PKA within the amygdala can lead to an augmentation of anxiety-like behavior, resulting in changes in amygdala activation in response to a gaze [93]. These findings confirm the amygdala’s role in encoding the perceived threat level of faces, as well as the more general assumption that the amygdala encodes the relevance or significance of a stimulus to the observer [94]. These results suggest that individual anxiety levels may explain inconsistent findings from previous studies [95]. Straube and colleagues [83] investigated whether the amygdala is activated by a gaze in response to threat relevance or facial expression by testing its activation during the observation of neutral, happy and angry faces with either a direct or averted gaze. An averted versus direct gaze enhanced amygdala reactivity, regardless of the emotional facial expression, suggesting that the amygdala has a role in gaze processing, despite the valence of the facial expression (as suggested in an earlier study in monkeys [96]). On the other hand, STS displayed an increased activation due to averted emotional versus averted neutral faces, even though this difference was not seen during a direct gaze, in line with the idea that emotional averted faces might be considered more strongly than neutral averted faces because they may have a greater influence on proper behavior in (ambiguous) social interactions. 

A potential confounding factor in the abovementioned studies is the use of static stimuli, which may have concealed the role of the amygdala. Therefore, Sato and colleagues [65] showed dynamic and static faces with angry or happy expressions. When dynamic stimuli were used, happy and angry faces with a direct gaze toward the subject caused higher amygdala activity than those looking away. Instead, when static stimuli were presented, a direct gaze had no effect on amygdala activation; only a trend for decreased amygdala activity was observed in response to facial expressions directed toward the subjects than for those directed away. In the same study, the STS was found to be more active in the contrast between dynamic and static stimuli, but not sensitive to the perception of emotion or gaze, in line with its role in perceiving biological motion [65].

In another fMRI study using an emotion recognition task [84], the authors investigated the neural networks underlying emotion recognition when a gaze is at play. Faces represented happiness, anger or a neutral expression with their gaze shifted toward their left or their right. The results showed the stronger connectivity of the STS and mPFC with both amygdalas while viewing an angry face with averted gaze than with a direct gaze, suggesting that gaze perception is not mediated by an encapsulated module, but emotional content can modulate the neural correlates of gaze perception. The idea of the involvement of STS in gaze processing was supported by a 7 T high-resolution fMRI experiment [85], which found a well-defined topographical subdivision within the STS, where the posterior and superior areas are related to gaze activity, while the middle and inferior regions are related to emotion. Finally, a recent study [86] replicated previous findings by demonstrating that the amygdala is activated by threatening emotional facial expressions and that it is highly responsive to direct gaze, but only in real faces and not in computer-generated faces. In addition, only the STS, and not the amygdala, responded differentially to gaze direction according to facial expression.

Overall, these results indicate a complex pattern of amygdala responses to gaze direction, which depends on individual differences, the type of emotion expressed by the face and gaze direction (see Table 1 for more details). Most of the aforementioned studies tested the role of the amygdala during averted and direct gaze only when threatening facial expressions were presented, preventing the possibility of investigating possible differences in emotion. However, in most of these studies, the elicited activity in the amygdala revealed a strong association between gazing and facial expression, suggesting its role in integrating the two sources of information, although not always in the same way. On the other hand, the human STS tends to be less responsive to the emotional expression of a facial stimulus but shows high sensitivity to gaze direction.

### 4.1. The Time-Course of Processing of Emotional Expressions and Gaze Direction in the Amygdala and STS

Magnetoencephalography (MEG) was also used to investigate the conjoint processing of emotion and gaze direction [97]. Lachat and colleagues [98] used MEG to assess whether combining averted gaze and a fearful facial expression could induce a differential early effect of attentional orienting response, in line with several EEG results that showed that fearful expressions were able to modulate early brain components (P1, N170 and N190) [99,100,101,102]. Lachat and collaborators found that averted gaze associated with a fearful expression led to an early attentional orienting effect only for congruent stimuli following fearful gaze cues, which was reflected at the neural level with the activation of the left superior parietal and left lateral middle occipital regions. No gaze-cueing effects were observed for the objects following happy gaze cues, and no evidence of the involvement of STS was reported [98]. However, these findings should be considered cautiously due to the limitations in the source localization of the MEG signal.

A subsequent MEG study [103] focused on the time course of the role of the amygdala in emotion and gaze perception, demonstrating that its activity was enhanced at an early stage for fearful compared to neutral faces (between 130 and 170 ms), while an effect of gaze direction in fearful faces was found at a later stage (between 190 and 350 ms). Moreover, a combined EEG and fMRI study [104] demonstrated that at earlier stages (170 ms), the amygdala processes the emotional information separately, while directional cues (gaze direction with pointing gesture) were integrated at approximately 190 ms in the parietal and supplementary motor cortices and not in the emotional system.

### 4.2. Transient or Sustained Presentation of Visual Stimuli

Speed processing represents a still unexplored element of threat perception. Most of the aforementioned studies [33,50,79] employed rapid presentation of visual stimuli, promoting visual input via the magnocellular pathway. This pathway is involved in immediate and quick orienting responses to danger [105]. Instead, Adams and colleagues [32] used prolonged (sustained) presentation of threatening faces, which favors the processing of visual information via the parvocellular system. Therefore, in a new study, the same authors [106] varied the presentation speed of fear displays. Interestingly, they found that responses of the amygdala to averted versus direct gaze in fearful expressions changed according to the presentation time: direct fearful gaze leads to increased left amygdala activity compared to averted fearful gaze when using longer presentation durations, replicating their original pattern of findings [32], while with a shorter presentation, a greater response was found for averted compared to direct fearful gaze (as in [33]).

These results hint at the presence of a fast and reflexive amygdala activation linked to obvious threat and a subsequent reflective activation linked to ambiguous threat (see also a subsequent study investigating sex-related differences in amygdala activation [107]). These findings were confirmed and expanded by a subsequent study from the same authors [97], which also found a higher phase locking to averted-gaze fear in the early response of the right amygdala, similarly to the initial connectivity between the left amygdala and orbitofrontal cortex. This finding suggests the rapid deployment of visual resources to congruent threat cues, and thus supports the right amygdala’s role in the immediate recognition of emotionally relevant stimuli. 

In the same study, the pSTS showed a different pattern of activation, with stronger responses for the short presentation of averted-gaze fear and the longer presentation of direct-gaze fear. The pSTS was involved in the late stages of longer stimuli presentations, highlighting its potential role as a constituent of the ‘social brain’ [108]. The ‘social brain’ is conceived as a core area in the visual integration of social cues [109], particularly when deducing the social meaning from both expressions and eye gazing [110], making it a likely candidate to solve the ambiguity of an incongruent threat cue during longer presentations.

### 4.3. Gaze Direction and Emotional Expression: Low-Level Visual Features

It is still unknown whether previously observed effects of gaze direction on amygdala activation depended to some degree on low-level features in the stimulus display (e.g., increase in visible eye sclera in the averted gaze conditions). Therefore, Hardee and colleagues [34] performed an fMRI study monitoring amygdala activation while participants observed fearful, gaze-shifting, happy and neutral eyes. They observed greater activity in the left amygdala only for the fearful eye observation. However, a condition combining fear and gaze was absent here since this issue was beyond the scope of the present work. Subsequently, Sauer and co-workers [111] included head direction manipulation in order to control for possible low-level confounding factors. Their data showed that faces with an averted gaze enhance amygdala activation more than faces with a direct gaze, despite the facial expression (angry, happy or neutral) or head orientation, suggesting that the amygdala plays an important role in attention processes and that averted gaze serves as a signal of socially relevant information. These findings support the study by Gamer and Büchel [112], which showed that amygdala activation was significantly enhanced when fearful faces were presented with the mouth aligned to fixation; therefore, when eye movements were directed to the eye region, this highlighted this area’s function in reflexive gaze initiations in response to salient stimuli rather than being driven by simple low-level features in a stimulus display.

## 5. Subliminal vs. Supraliminal Cues

It is possible to trigger attentional orienting with eye gaze information even when such information is subliminal and participants are not aware of it, as demonstrated by multiple studies [113,114,115,116]. These studies propose that subliminal and supraliminal gaze cues can automatically foster attentional orienting and may, therefore, share the same neural circuit [117,118]. To address this issue, in 2016, Sato and colleagues [119] assessed brain activity using event-related fMRI while participants watched averted or straight-ahead gaze cues, displayed in supraliminal or subliminal conditions. The gaze cue was presented for 13 ms and then covered by a mask in the subliminal condition, while in the supraliminal condition, the gaze cue was shown for 200 ms without a mask. In both supraliminal and subliminal conditions, a gaze-cueing effect was reported (i.e., reaction times were faster when gaze cues were congruent with object locations than when they were incongruent), consistent with previous findings [120]. Widespread cortical activity was observed for both the supraliminal and subliminal presentation of stimuli with averted eyes compared to straight eyes. The middle temporal gyrus (including the superior temporal sulcus), inferior parietal lobule, anterior cingulate cortex, superior and middle frontal gyri and superior and middle frontal gyri were the most active regions. These findings suggest that the temporo-parieto-frontal attentional network is implicated in both conscious and unconscious gaze-triggered attentional shifts.

## 6. Brain-Damaged Patients and Transcranial Magnetic Stimulation (TMS) Evidence

**Figure 2:**
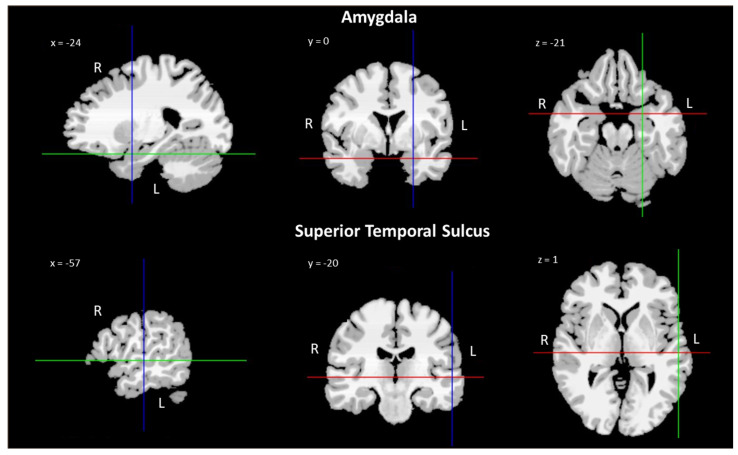
Neuroanatomic MRI images of the amygdala and superior temporal sulcus (STS) with MNI coordinates. The slices of the brain in the different axes of the present figure were generated free of charge using Bioimage Suite Web (https://bioimagesuiteweb.github.io/webapp/mni2tal.html, accessed on 24 February 2022).

Recent growing evidence suggests that non-invasive brain stimulation techniques (NIBS) can be used to induce neuroplasticity in order to modulate cognition and behavior [121,122], unveiling the critical role of specific neural circuits [123,124]. Therefore, NIBS might be applied to modulate low-level mechanisms that mediate, for example, attentional orienting to gaze cues, highlighting the functional role of brain areas (i.e., the amygdala and STS) in the gaze-cueing effect (see Figure 2) [21,125,126,127].

Direct evidence for the roles of the STS and amygdala in gaze processing can also be derived from brain-damaged patients. Two studies published in 2006 [128,129] reported the case of a patient with a critical deficit in gaze perception following a stroke in the right STS. Similarly, findings were reported in non-human primates with temporal ablation in which STS damage resulted in an impaired recognition of different gaze angles [88]. For instance, a reversible deactivation (i.e., muscimol injections) of the pSTS in monkeys suppressed gaze-cueing behavior [130]. In addition to STS lesions, it was also reported that amygdala lesions impair attention to eye information and averted gaze-cueing effects [8,26,31,131,132,133]. Spezio and colleagues [133] demonstrated that amygdala lesions induce longer gazing at the mouth region compared to the eye region during a real conversation, suggesting that the amygdala is involved in recognizing and processing important social cues and directing attention accordingly [8,31]. Finally, an investigation on the interaction involving emotion and gaze processing in amygdala patients [132] demonstrated that a unilateral lesion in the right amygdala affects the interaction of gaze and expression during emotion perception. Thus, this finding adds support to recent theories that the human amygdala might be essential for encoding the self-relevance of emotional events. In particular, amygdala patients showed deficiencies in the effect of direct gaze on the perception of angry expression, implying that gazing plays a greater role in the interpretation of anger.

Although many studies have focused on the critical role of the STS in emotion perception [134,135,136], a limited number of human studies used non-invasive transcranial magnetic stimulation (TMS) to target pSTS and influence gaze processing [137,138]. Saitovitch and colleagues [138] administered inhibitory repetitive TMS (i.e., continuous theta-burst stimulation) over the right STS and found significantly fewer fixations toward someone else’s gaze while viewing social scenes, thus suggesting a top-down effect of the STS on social attention (deploying attentional resources to the eyes and lips of the actors in the presented social scenes). In another TMS study [137], the authors applied single-pulse TMS to investigate the critical role of STS in a gaze direction task. The authors found that TMS applied over the STS 100 or 200 ms after the presentation of a second matching face stimulus (the same/different to the first face gaze direction) hampered the perception of gaze shift. This demonstrates the critical and causal function of the STS at early latencies in extrapolating gaze-shift information. Indeed, results showed that TMS impaired responses exclusively in trials in which there was a gaze shift between the first and the second presentation of faces in pairs (i.e., from straight to averted gaze or vice versa), but TMS had no effect on responses during trials with static gaze positions (straight–straight and averted–averted pairs). These findings suggest that TMS effects during gaze processing were notably content specific, primarily affecting the perception of gaze shifts instead of static eye positions. Together, the lesion and TMS studies suggest a critical and causal role of the STS at early latencies in the process of extrapolating gaze direction information.

## 7. Conclusions

In this review, we addressed the neural bases of the perceptual processing of the direction of another’s eye gaze and the gaze-cued attentional orienting process. In addition, we reviewed modulation by contextual factors on the neural bases of gaze direction and gaze-cueing, such as the kind of cue that induced attention orienting (i.e., gazing or non-symbolic cues) or the emotional expression conveyed by the gaze cues. We reported many inconsistencies between the reviewed findings, which prompt further research in this area. Such inconsistencies may be explained by the different methods employed, such as task instruction (i.e., passive observation vs. gaze discrimination), type of emotional expressions (i.e., fear vs. anger as negative stimuli), kind of facial stimuli (i.e., static vs. dynamic) and scanning parameters. All these methodological differences represent a limit when results from different studies need to be compared. For example, N’Diaye and colleagues [79] demonstrated that the emotional intensity of the presented facial expression is able to interact with the gaze effect. Thus, the absence of systematic manipulation of the emotional intensity may represent a limit in the experimental design. Another example was reported by Zhao and colleagues [72], who proposed that attentional orienting differs between eye gaze and arrow cues when these cues are influenced through contextual processing. Similarly, the context seems to be able to modulate the activity of STS. Thus, choosing to add contextual information or not may dramatically impact both the behavioral and neuroimaging results. However, from the reviewed studies, we extracted the following key take-home messages.

First, we concluded that although several authors reported a specific function of the STS in processing gaze cues compared to non-symbolic cues [42,62,64,70], multiple studies questioned the specificity of the STS for the perception of gaze cues, preventing us from ascertaining with confidence whether the human STS is specialized for attentional orienting to social stimuli [68,69,72,75].

Second, when emotional expressions are presented, the STS seems to have a pivotal role in gaze perception and to be less sensitive to emotions, while the amygdala seems to play an important role in the interaction between gaze and emotional expressions, especially when negative expressions are presented [32,33,42,79]. This negative advantage is not surprising since it was demonstrated that faces expressing fear and anger are subject to a distinct processing advantage, which leads to them being analyzed quickly by the brain (~120 ms) akin to the way in which faces are structurally encoded [139,140,141], and evoking fast autonomic responses [142,143,144,145]. Studies measuring RTs have also revealed how faces expressing negative emotions enhance rapid and involuntary attention to the stimulus [146,147]. Moreover, negative expressions rapidly impact the corticospinal excitability, as measured with motor evoked potentials [148,149,150,151,152,153,154]. However, despite the clear importance of interpreting emotional expression and their direction of gaze for social communication, only a few studies reported increased STS activation for emotional expression (also positive) and an averted versus direct gaze [81]. 

Finally, few studies have found the amygdala to be activated for the averted gaze, regardless of facial expression [83], suggesting that STS possibly codes individual gaze directions [56], while the amygdala could play a specific part in perceiving or monitoring eye contact [45,46], possibly reflecting an emotional response to being looked at [155]. More effort is required to disclose the interplay between socially relevant stimuli and the information conveyed by gaze cues.

Together, the available neuroimaging results on the contextual (i.e., head orientation) modulation of gaze, especially the proposed increased invariance to gaze-independent characteristics from posterior to anterior STS [60], paves the way for questions such as whether and how social variables could potentially be processed in the framework of gaze perception. Carlin and Calder [156] suggested that gaze processing requires a large network of brain regions that includes anterior and posterior sections of the STS, the lateral parietal cortex and the medial prefrontal cortex. In particular, they suggested that the anterior STS may be important in the perceptual processing of gaze because it discriminates between different averted gaze directions in an approach that is invariant to head view and physical image features [60]. The preferential role of the anterior STS in view-invariant representations of gazing reveals a hierarchical processing stream for gaze perception, with greater invariance to gaze-independent features (e.g., head orientation) from the posterior to anterior STS. Indeed, the lateral parietal cortex, and the posterior part of the STS in particular, may contribute to gaze-cued attentional orienting, which is influenced by physical stimulus features [64]. Carlin’s findings raise an interesting finding: on the one hand, emotional context, social status and familiarity conveyed by physical visual features could be filtered out in the posterior part of the STS before reaching an invariant representation that is useful for gaze perception in the anterior STS. On the other hand, some of these contextual variables (i.e., emotional context, social status and familiarity) could instead become an integral part of the invariant representation and be encoded in the high-order (supra-modal) processing of gaze-cueing that occurs in the anterior STS; however, future studies are needed to address this point. Taken together, previous imaging studies converge in suggesting a role of STS in attentional orienting, even when gaze information is subliminally presented, which is less sensitive to emotions, while the amygdala seems to play a role in jointly elaborating gaze and emotional expressions. These results are supported by brain-damaged patients and neuromodulation studies, which reported deficits in gaze perception and gaze-cueing behavior after lesions or the temporal inactivation of the STS. Similarly, amygdala lesions impair attention to eye information and averted gaze-cueing effects. Moreover, the unilateral lesion in the right amygdala affects the interaction of gaze and expression during emotion perception. However, the heterogeneity of the stimuli and paradigms employed indicate that the exact role of the two structures when multiple stimuli need to be processed is yet to be elucidated.

## Figures and Tables

**Table 1:** Summary of neuroimaging findings in studies with emotional facial expressions in different gaze direction paradigms.

Study	Emotional Facial Expression	fMRI Contrast	Paradigm	Coordinates (MNI)	Main Findings
Hooker et al. (2003)	Happy–angry	Angry > happy	Gaze-cueing	Right STS: 42 −57 26 Left STS: −35 −67 31	STS for angry faces > happy faces
Adams et al. (2003)	Fear–angry	Angry averted/fearful direct > angry direct/fearful averted	Passive observation	Left amygdala: −15 0 −18	Amygdala > angry averted and fearful directed gaze
Sato et al. (2004)	Angry–neutral	Expression × face direction	Gender task	Left amygdala: −22 −9 −16	Amygdala > angry expressions looking toward
Engell and Haxby (2007)	Neutral direct–averted gaze anger, disgust, fear and surprise with direct gaze	Emotions > neutral Averted > direct	Match identity task	Right STS (emotion): 52 −48 8 Right STS (gaze): 36 −54 15	STS emotions > neutral faces STS averted > direct gaze faces
Hadjikhani et al. (2008)	Fear	Fearful averted > fearful direct	Passive observation	Right STS (gaze): 48 −54 14 Amygdala (emotion): −12 −11 26	Amygdala and the STS fearful averted gaze > fearful direct gaze
N’Diaye et al. (2009)	Fear–angry (intensity morphing)	Emotions > neutral Averted > direct	Emotional intensity rating	Left amygdala (emotion): −24 +3 −18 Right amygdala (emotion): +24 +6 −15 Left pSTS (gaze): −57 −42 +9 Right pSTS (gaze): +45 −66 +12	Amygdala fearful averted gaze > directed gaze Amygdala angry directed gaze > averted gaze STS averted gaze > direct gaze
Ewbank et al. (2010)	Fear–angry–neutral	Angry direct > neutral direct Angry direct > angry averted Fearful averted > neutral averted	Gender task	Right amygdala (gaze): 22 −6 −12 Right amygdala (emotion × gaze): 24 −4 −12	Amygdala high-anxious angry direct gaze > angry averted gaze Highly anxious > fearful direct gaze and averted gaze
Straube et al. (2010)	Happy–angry–neutral	Averted > direct Valence × gaze	Gender task	Left amygdala (gaze): −27 −9 −14 Right amygdala (gaze): 16 −6 −11 Right STS (valence × gaze): 54 −49 16	Amygdala averted gaze > direct gaze STS averted emotional > averted neutral
Sato et al. (2010)	Happy–angry (static and dynamic)	Dynamic > static Direct > averted	Gender task	Left amygdala: −18 −10 −10 Right STS: 58 −46 8	Amygdala dynamic happy and angry directed gaze > averted gaze STS dynamic > static
Krämer et al. (2014)	Happy–angry	European angry averted > Asian angry averted	Valence rating	Right amygdala: 28 −8 16	Amygdala > angry averted gaze in ingroup
Ziaei et al. (2017)	Happy–angry–neutral	Expression with averted > all other conditions	Emotion recognition task	Left STS: 56 6 2	Stronger connectivity of the STS and mPFC with bilateral amygdala for angry averted gaze > direct gaze
Schobert et al. (2018)	Happy–angry (gaze shift and speech motion)	Emotional (happy and angry) > non-emotional (gaze and speech) Eyes (angry and gaze) > mouth (happy and speech)	Identity detection task	Left pSTS: −50 −48 15 Right pSTS: 50 −47 13	pSTS gaze-happy and gaze-angry > speech-happy and speech-angry
Kätsyri et al. (2020)	Angry–fear–neutral	Directed > averted + angry > fear	Circle detection in catch trials	pSTS: 65 −54 3	Amygdala direct gaze > averted gaze Amygdala threatening > neutral STS gaze direction × emotional expression

## Data Availability

Not applicable.
